# Data on the diagnosis of the management of the primary waste from electrical and electronic equipment in health care institutions in Barranquilla, Colombia

**DOI:** 10.1016/j.dib.2020.106236

**Published:** 2020-08-27

**Authors:** Helen Marcela Gandara-Perez, Nelson Enrique Lubo-Hoyos, Samir Fernando Castilla-Acevedo, Fabio Armando Fuentes-Gandara

**Affiliations:** Natural and Exact Sciences Department, Universidad de la Costa, Calle 58 #55 – 66, 080002 Barranquilla, Colombia

**Keywords:** WEEE, Health care institution, Biomedical equipment, Collection centre, Final disposal, Management

## Abstract

Economic and technological development advances exponentially, and the implementation of new technologies in the health sector has become a source of waste for electrical and electronic equipment (WEEE). Electrical and electronic equipment must be replaced periodically, either due to a technological update or to improve medical treatments, which ultimately leads to the generation of this type of waste. This work aimed to conduct exploratory research on the current situation of the handling of this type of waste in Barranquilla, Colombia, considering the limited information related to the management of biomedical WEEE in the city. Sixty health care institutions (HCIs), including hospitals and clinics, odontological centres and ophthalmological centres, participated favourably in the surveys concerning the management of WEEE. Through this work, it was possible to establish a working staff level of knowledge on WEEE disposal and the framework for the management and temporary collection of this waste. Therefore, the data are useful for proposing strategies for the integral management of electrical and electronic waste in both small and large populations.

## Specifications Table

**Table:** 

Subject	Waste management and disposal
Specific subject area	Solid waste collection and disposal
Type of data	Tables and figures
How data were acquired	The data were acquired through an on-site questionnaire answered by the person in charge of solid waste management at 60 health care institutions.
Data format	Raw and analysed data
Parameters for data collection	The questionnaire consists of 15 main questions that allow the identification of the main WEEE generated in the medical centres of Barranquilla, Colombia, as well as information related to the management and the final disposal that HCI gives to WEEE when they have fulfilled their use.
Description of data collection	The data collection is the result of a standardised and structured questionnaire administered to 60 health care institutions located in Barranquilla, Colombia. The institutions were divided into hospitals and clinics, odontological centres and ophthalmological centres.
Data source location	City: Barranquilla Country: Colombia
Data accessibility	Dataset is available within the data article as a supplementary file and on Mendeley data repository: https://data.mendeley.com/datasets/st5g3fw8bx/2

## Value of the Data

• To the best of our knowledge, this is the first analysis of the management and final disposal of WEEE in the city of Barranquilla. Therefore, this study will help stakeholders evaluate existing policies, understand the implications of future compliance, identify the challenges and opportunities related to efficient resource recovery from WEEE and support the transition towards the improvement of the comprehensive management of WEEE through a new set of legislative requirements.
• The data allow us to know about the HCIs that are unaware of their obligations regarding the proper use, collection, and management of WEEE established by policymakers and other stakeholders.
• This type of data allows environmental authorities to monitor clinics and odontological and ophthalmological centres to assure the improvement of their integral management of WEEE, thereby achieving the requirements established either in the national or international technical guidelines.
• The data also provide interested parties, such as academics, those in the health sector, and local and national authorities, with a baseline for decision-making actions focused on research about WEEE wastes and normative and technical contributions, among others.

## 1 Data Description

The raw data about WEEE management collected through the questionnaire (see supplementary material) are presented in Tables 1–9. Table 1 indicates the number of health institutions that reported possessing essential biomedical equipment considered as a type of WEEE. Table 2 shows the number of HCIs that selected some non-biomedical devices, such as electrical and/or electronic devices. Table 3 shows the number of HCIs who stated that they know the Colombian legal regulations for WEEE management. The estimated amount of WEEE reported by hospitals and clinics is reported in Table 4, and the number of HCIs that have a storage centre for WEEE is shown in Table 5. The information collected on the types of storage of these wastes, the difficulties presented for their management and how their final disposal is carried out are presented in Tables 6, 7 and 8, respectively. Finally, Table 9 shows the amount of WEEE stored by the different HCIs.

**Table 1:** Number of health care institutions that selected biomedical-type electric and electronic equipment. H&C: hospitals and clinics; ODC: odontological centres; OPC: ophthalmological centres.

Equipment	Institutions type	Number of institutions
Vital sign monitor	H&C	40
Centrifuge	H&C	32
X-ray scanner	H&C	40
Microscope	H&C	26
Digital thermometer	H&C	40
Freezer	H&C	32
Electrosurgery	H&C	34
Electrocardiograph	H&C	40
Defibrillator	H&C	40
Incubator	H&C	28
Autoclave	H&C	34
Infusion system	H&C	40
Anaesthetic vaporizers	H&C	38
Ultrasound machine	H&C	28
Mechanical ventilator	H&C	34
Pulse oximeter	H&C	40
Doppler foetal monitor	H&C	28
Medical scales	H&C	38
Wrist monitor	H&C	40
Medical lamps	H&C	40
Serological bath	H&C	32
Haematological equipment	H&C	36
Suction unit	H&C	40
Anaesthesia machines	H&C	40
Dental unit	ODC	10
Dental compressor	ODC	10
High-low speed handpiece	ODC	10
Apical locator	ODC	6
Dental and panoramic radiology	ODC	8
Autoclave	ODC	8
Curing light	ODC	10
Dental ultrasound machine	ODC	8
Saliva and blood suction equipment	ODC	10
Dental amalgamator	ODC	4
Dental anaesthesia	ODC	10
Ophthalmologic projector	OPC	8
Pupillometer	OPC	8
Tonometer	OPC	10
Lensometer	OPC	10
Keratometry	OPC	10
Slit lamp	OPC	10
Indirect ophthalmoscope	OPC	10
Retinoscope	OPC	10
Ophthalmoscope	OPC	10

**Table 2:** Number of health care institutions that selected non-biomedical-type electric and electronic equipment.

Equipment	Hospitals and clinics	Odontological centres	Ophthalmological centres
Medical lamp	34	10	10
Lamps and LEDs	38	10	10
Computer	40	10	10
Electrical and electronic tools	22	4	6
Devices with screens	40	10	10
Small domestic appliances	30	2	2
Roof lamps	40	10	10
Diagnostic equipment	32	4	8
Other	14	0	0

**Table 3:** Number of respondents who know or do not know any Colombian legal regulations for WEEE management in this type of health care institution.

Answer	Hospitals and clinics	Odontological centres	Ophthalmological centres
Yes	20	2	6
No	20	8	4

**Table 4:** Amount estimated of WEEE generated per year by clinics and hospitals.

Range of amount generated	Number of hospitals and clinics
0-20	0
21-40	0
41-80	10
81-120	4
121-150	14
Another amount	12

**Table 5:** Number of health care institutions that have a collection or storage centre.

Answer	Hospitals and clinics	Odontological centres	Ophthalmological centres
Yes	28	2	6
No	12	8	4

**Table 6:** Storage types given to WEEE by HCIs.

Type of storage	Number of hospitals and clinics	Number of odontological centres	Number of ophthalmological centres
Containers	12	2	2
Open air	14	0	1
Plastic bags	12	5	5
Cardboard boxes	3	2	3
Special plastic	8	0	2

**Table 7:** Difficulties in the WEEE storage generated in HCIs.

Difficulties	Number of hospitals and clinics	Number of odontological centres	Number of ophthalmological centres
Little space to store	17	10	5
Carrying equipment	14	3	5
Size of the equipment	9	2	0
None	9	0	3
Brittleness of equipment	3	0	0

**Table 8:** Different final disposals available for WEEE.

Type of final disposal	Number of hospitals and clinics	Number of odontological centres	Number of ophthalmological centres
Return to suppliers	18	4	5
Parts reuse	17	5	5
Donation	17	5	5
Scrapping	15	0	0
Post-consumption plan	13	4	3
Equipment sales	11	3	4
Cleanliness operator	3	0	1
Recycling	3	3	1

**Table 9:** Amount and type of WEEE stored (biomedical or non-biomedical.

Amount	HCI with biomedical WEEE stored	HCI with biomedical WEEE stored
Currently empty	44	38
Not sure	4	4
From 1 to 10	6	8
From 11 to 20	2	2
From 31 to 40	4	2
From 41 to 50	0	6

**Fig. 1:**
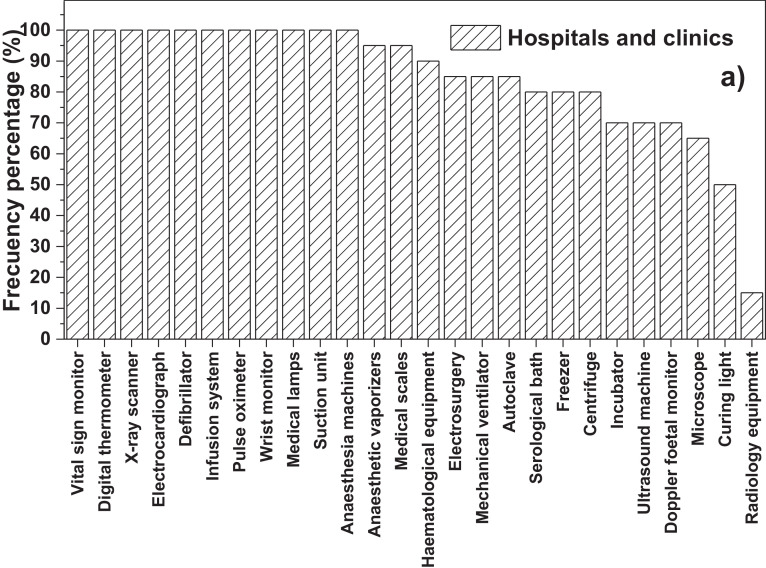

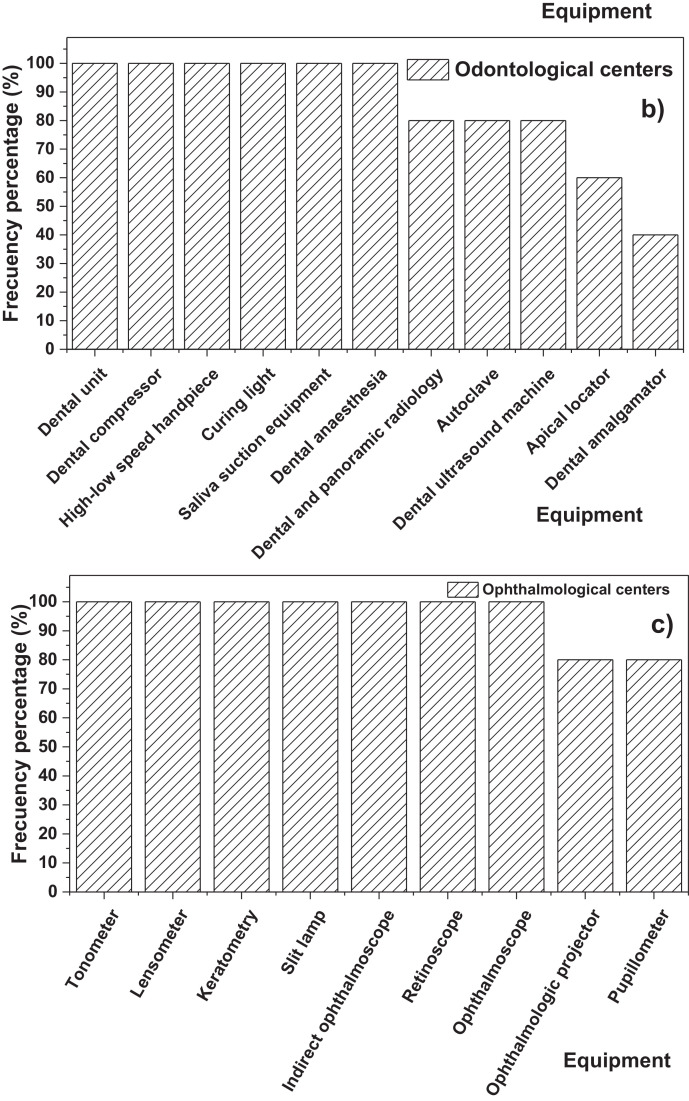
Types of biomedical-type electric and electronic equipment for medical diagnoses and procedures. a) Hospitals and clinics. b) Odontological centres. c) Ophthalmological centres.

**Fig. 2:**
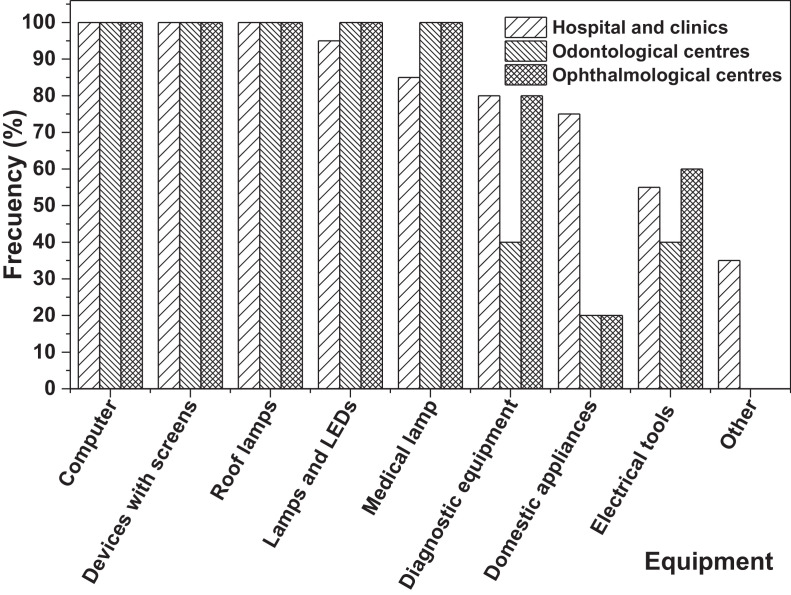
Types of non-biomedical electrical and electronic equipment in HCIs.

**Fig. 3:**
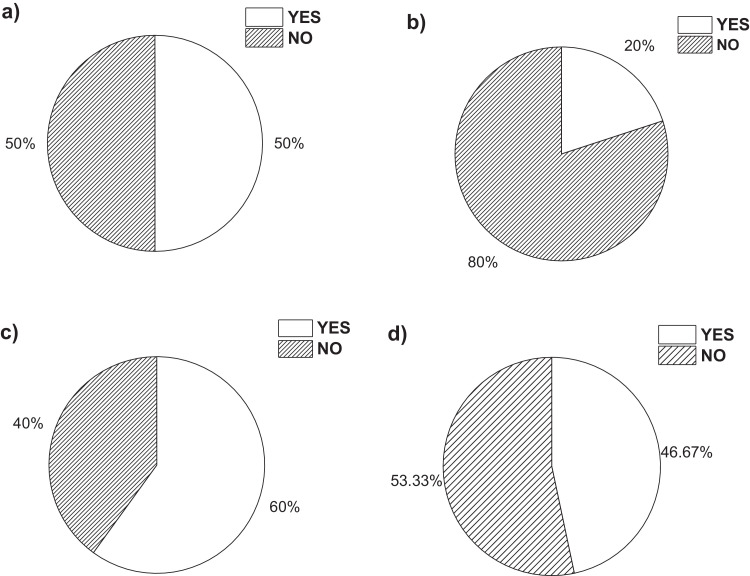
Percentage of respondents who either know or do not know any Colombian legal regulations for WEEE management in this type of health care institution. a) Hospitals and clinics. b) Odontological centres. c) Ophthalmological centres. d) Global data.

**Fig. 4:**
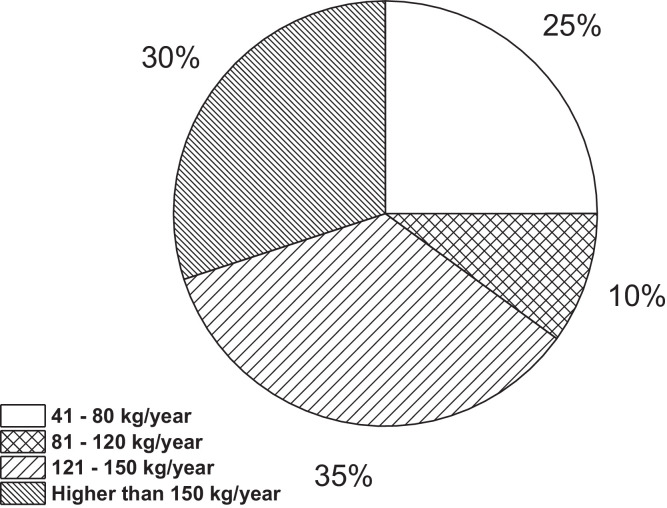
Amount of WEEE produced per year in hospitals and clinics.

**Fig. 5:**
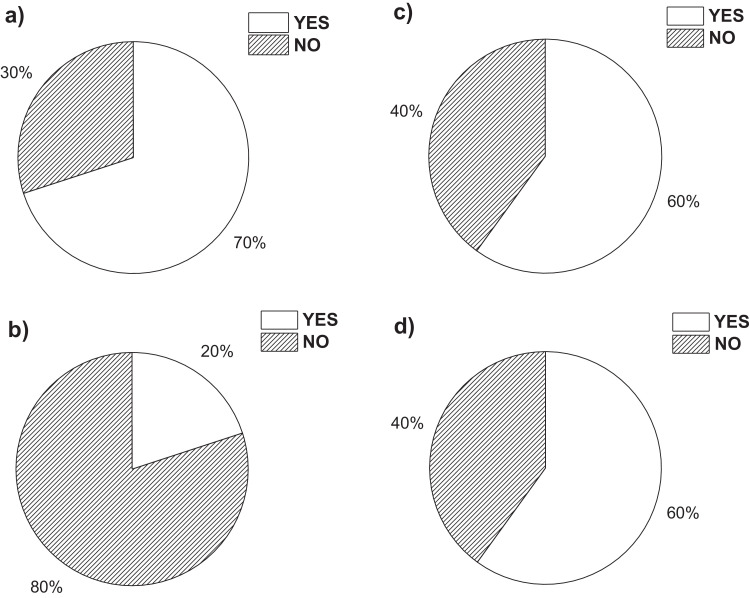
Percentage of HCIs that have a collection or storage centre. a) Hospital and clinics. b) Odontological centres. c) Ophthalmological centres. d) Global data.

**Fig. 6:**
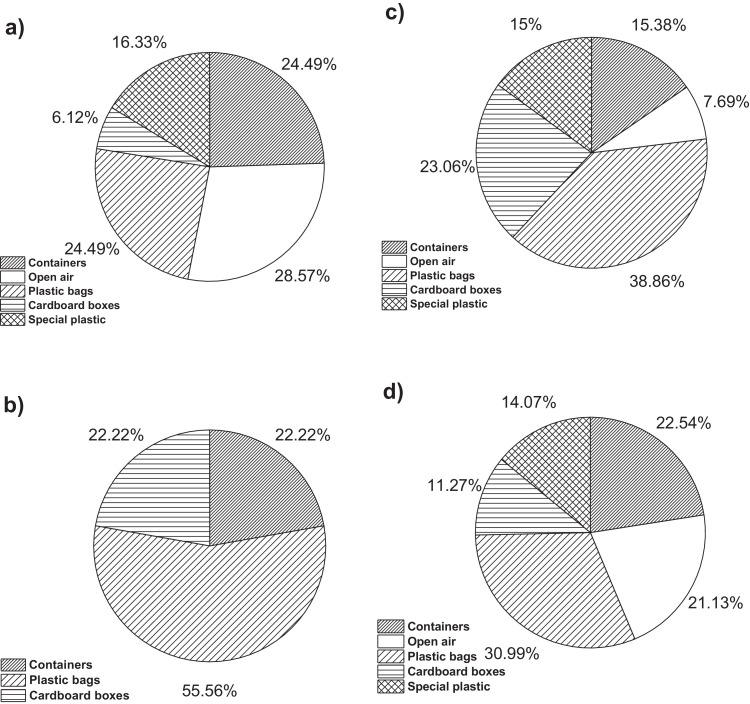
WEEE storage type in HCIs. a) Clinics and hospitals. b) Odontological centres. c) Ophthalmological centres. d) Global data.

**Fig. 7:**
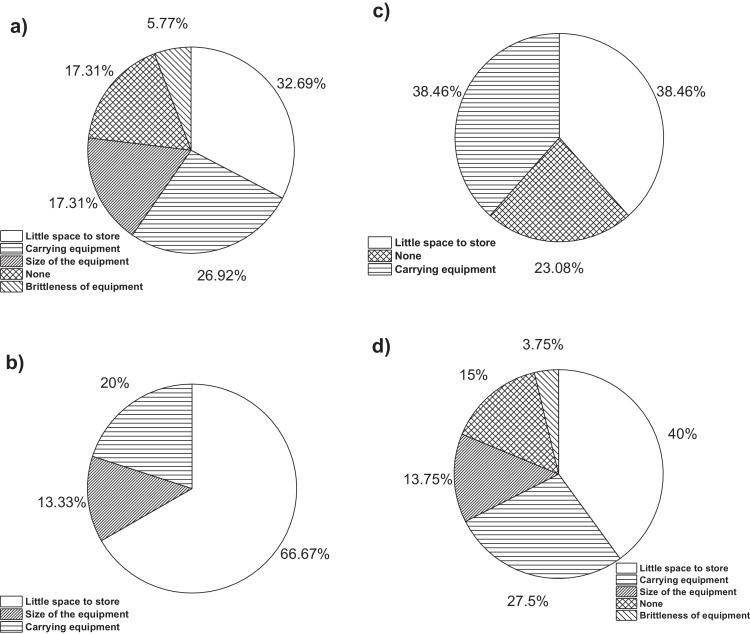
Difficulties in the WEEE storage generated in HCIs. a) Clinics and hospitals. b) Odontological centres. c) Ophthalmological centres. d) Global data.

**Fig. 8:**
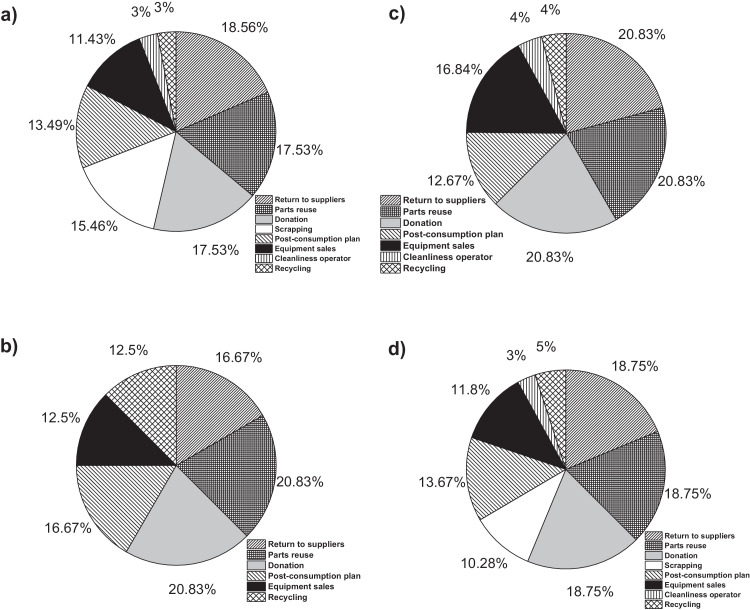
Final disposal for WEEE in HCIs. a) Clinics and hospitals. b) Odontological centres. c) Ophthalmological centres. d) Global data.

**Fig. 9:**
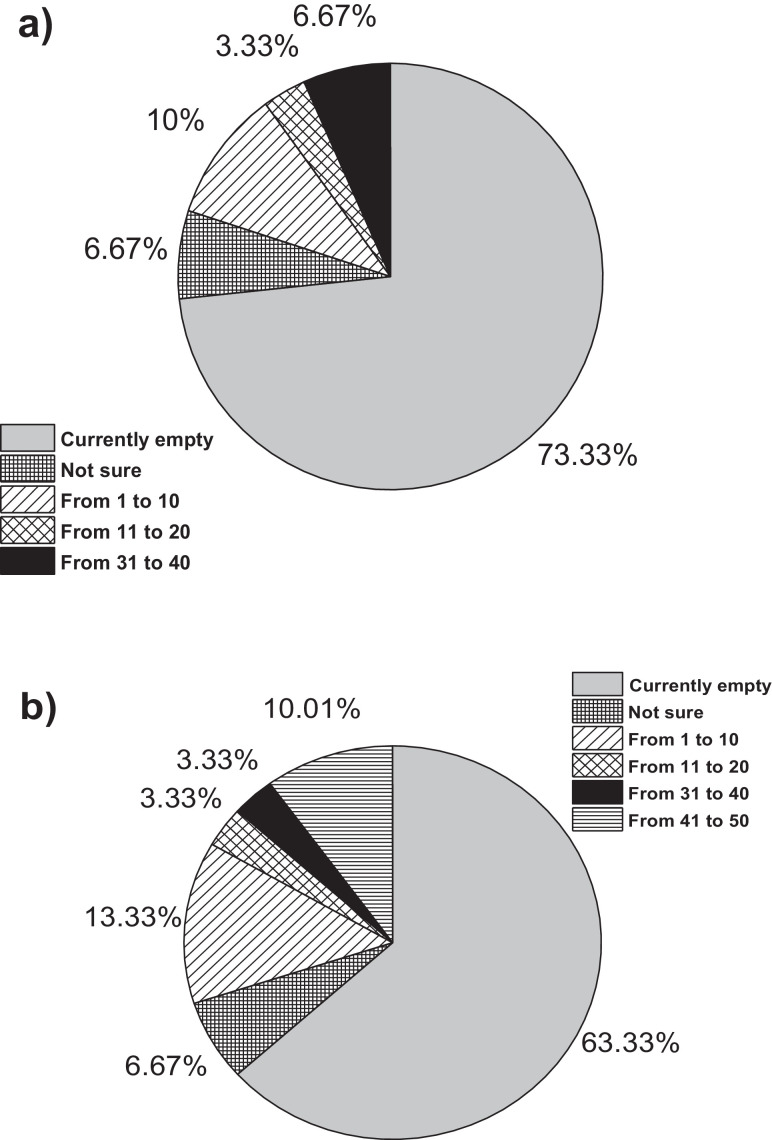
Total amount and type of WEEE stored. a) Biomedical. b) Non-biomedical.

**Fig. 10:**
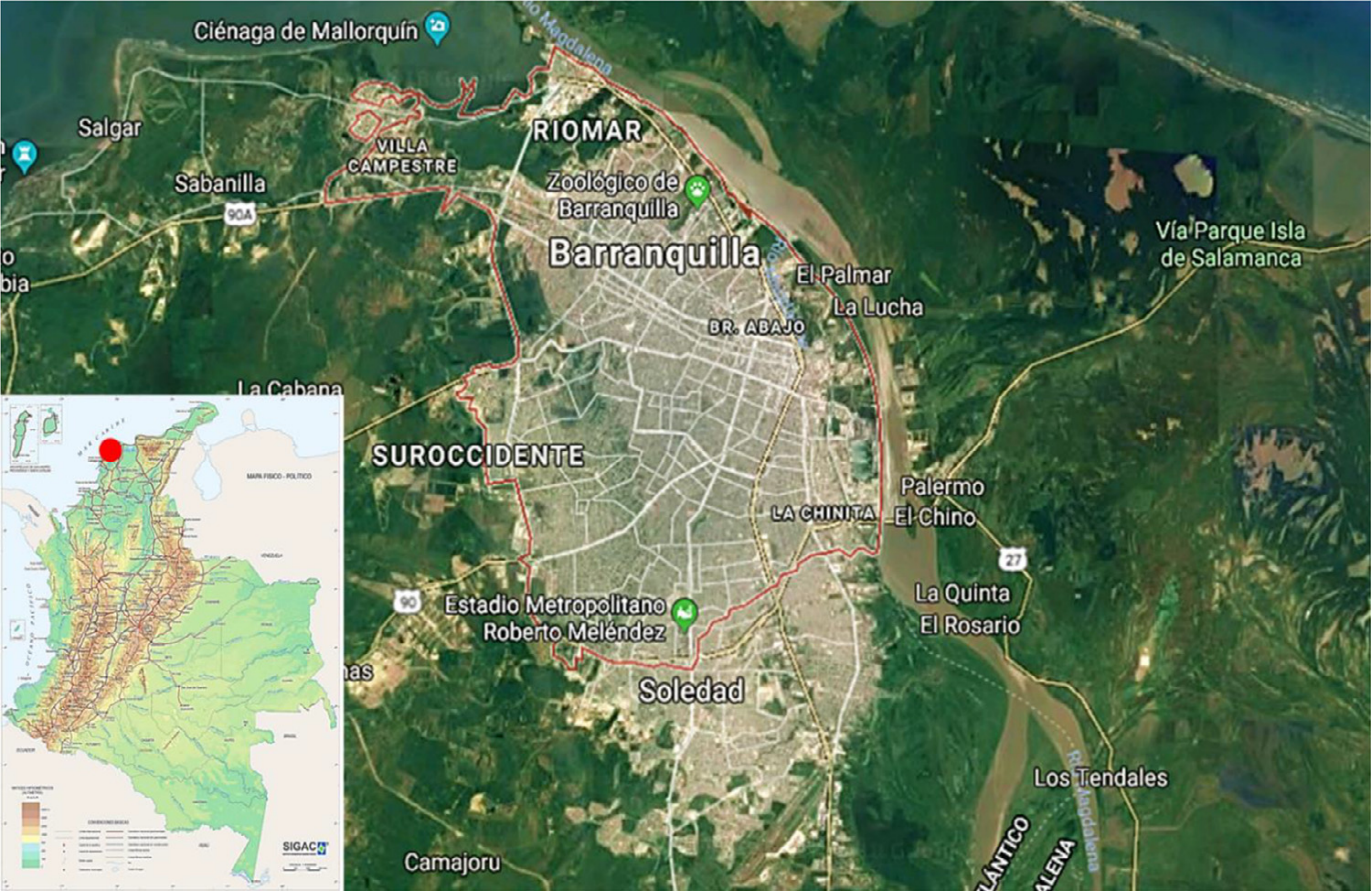
Geographic location of the study area. Source: Google Earth, 2018.

On the other hand, Fig. 1a**–**c shows the frequency percentages of biomedical-type electric and electronic equipment used in hospitals and clinics, odontological centres and ophthalmological centres, respectively. Additionally, Fig. 2 presents the non-biomedical type of equipment used in health care institutions. During the survey application, it was evident that at some HCIs, the interviewed personnel were doubtful when answering questions regarding WEEE. Therefore, it was decided to introduce a question about the management of electrical and electronic wastes. Fig. 3a***–***c shows the employees’ knowledge about the Colombian legal regulations for WEEE management in clinics and hospitals, odontological centres and ophthalmological centres, respectively, while Fig. 3d shows the overall percentage of employee knowledge in all the health care institutions surveyed. The amount of WEEE per year in hospitals and clinics is depicted in Fig. 4. For odontological centres and ophthalmological centres, this information is not reported since those HCIs answered that the annual amount of WEEE generated is not determined. However, those HCIs estimated an amount higher than 150 kg per year. Fig. 5a**–**d shows the information obtained from the survey related to the existence of collection or storage centres in HCIs. Fig. 6a–d presents the type of storage used for WEEE in the collection centres in hospitals and clinics, odontological centres, ophthalmological centres, and the global data of the HCIs surveyed, respectively. According to the information obtained from interviewed personnel, the main difficulties related to the temporary storage of WEEE generated in HCIs are shown in Fig. 7a–d. The final disposal of the WEEE generated in hospitals and clinics, odontological centres, ophthalmological centres, and HCIs in general is presented in Fig. 8a–d, respectively. Fig. 9a and b shows the total amount and type of WEEE stored (biomedical or non-biomedical), respectively. Finally, Fig. 10 shows the map of the city in which the study was carried out.

## 2 Experimental design, materials, and methods

### 2.1 Study area

The study was carried out in Barranquilla, which is located in northern Colombia, on the western part of the Magdalena River to 7.5 km from its mouth in the Caribbean Sea (Fig. 10). The city is in the humid tropical climate zone, with a latitude of 10° 59’ 17” to the north of Ecuador and a longitude of 74° 47’ 22” to the west of the Greenwich meridian. Barranquilla is also located approximately 1000 km to the north of Bogotá, the Colombian capital. The average altitude of Barranquilla concerning sea level is 18 metres above sea level, and its average temperature is 28 °C. The rains are generally manifested in torrential downpours. The relative humidity varies between 60% and 85% [1,2].

### 2.2 Type of research

Initially, an extensive review was carried out to determine the novelty of the research work. Then, the area and study population were delimited. An existing database located on the district health secretariat website showed a list of the health care institutions of the city. Therefore, this list was filtered according to the following criteria: 1) type of medical service offered (hospitals and clinics, odontological centres and ophthalmological centres; 2) the level of complexity (high and medium infrastructures) and 3) location in the Norte-Centro Histórico (North Historic Center) locality. The research nature was exploratory, considering that the generation and management of the waste from electrical and electronic devices of biomedical and non-biomedical origin in different HCIs is a non-common topic in the literature. The recollection of these data has the primary purpose of identifying the associated problems in clinics and hospitals, odontological centres and ophthalmological centres associated with the management of WEEE in health care institutions located in Barranquilla. It is worth noting that the survey was based on the main WEEE that are commonly used in these HCIs.

### 2.3 Population and sample

A population of 124 HCIs was selected, according to the information obtained from the district health secretariat of Barranquilla about the number of health care institutions that existed in 2016. These mainly correspond to clinics, hospitals, odontological centres and ophthalmological centres. These areas are located in the Norte-Centro Histórico locality. A letter of invitation was delivered to each of the HCIs to participate in the survey by answering a questionnaire designed for the collection of information. Of all the institutions invited to participate, only 60 contributed to the study and answered all the questions on the questionnaire. This value includes 50% of the total population of clinics and hospitals in the locality, as well as 50% of the ophthalmological centres and 42% of the odontological centres. It is also important to mention that the comprehensive management of WEEE is a matter of great discretion in the eyes of some entities providing health services. Therefore, not all HCIs are willing to collaborate with this type of study.

### 2.4 Information collection

The different health centres were surveyed between February and October 2018. A survey with 15 questions was applied (see the supplementary information material), and general information was obtained that allowed us to identify the main WEEE generated in the different HCIs of the city, as well as the management and final disposal that the HCIs give them when they have completed their use. The questionnaire was applied directly to the administrative personnel of solid waste management at each HCI. Therefore, it could be said that the quality of the information collected is comparable across the questionnaires. The questions were unambiguous, and there was no conflict of interest between the institutions that participated and the authors of this work. The different types of WEEE and the disposal and storage methods were selected based on previously reported research work in the city of Bogotá, Colombia [3]. All the questionnaires were fully completed by the personnel in charge of that type of residue and returned to the data collector. The questionnaire also included open-ended spaces to allow the participant institutions to add/include other items different from those given on the pre-existing list. The data were analysed through absolute and relative frequencies.

## Ethics statement

There were no ethical problems with the survey application or the collection of information since we worked with HCIs who voluntarily decided to participate in this investigation. In addition, there was no conflict of interest between the institutions and the authors of the study. The researchers ensured that the respondents were related to the administrative personnel in charge of managing the focal type of waste. Hence, these respondents could provide information with the highest possible reliability. Furthermore, the respondents were assured that their data would be treated confidentially.

## CRediT authorship contribution statement

**Helen Marcela Gandara-Perez:** Investigation, Formal analysis, Writing - original draft. **Nelson Enrique Lubo-Hoyos:** Investigation, Formal analysis, Writing - original draft. **Samir Fernando Castilla-Acevedo:** Software, Validation, Data curation, Writing - review & editing, Visualization. **Fabio Armando Fuentes-Gandara:** Conceptualization, Methodology, Supervision, Visualization, Writing - review & editing, Project administration, Funding acquisition, Resources.

## Declaration of Competing Interest

The authors declare that they have no known competing financial interests or personal relationships that have, or could be perceived to have, influenced the work reported in this article.

## Appendix Supplementary materials

Image, application 1

Image, application 2

Image, application 3

Image, application 4
