# Supplementary material for: Data on the diagnosis of the management of the primary waste from electrical and electronic equipment in health care institutions in Barranquilla, Colombia
Source: Data Brief. 2020 Aug 27;32:106236. doi: 10.1016/j.dib.2020.106236 (PMC7476238; doi:10.1016/j.dib.2020.106236)
Supplement: Supplementary file 1 [file mmc1.pdf]

Editing Certificate

This document certifies that the manuscript

Data on the diagnosis of management of the primary wastes of electrical and electronic equipment in the health care institutions in Barranquilla, Colombia

prepared by the authors

Helen Gandara-Perez, Nelson Lubo-Hoyos, Samir Castilla-Acevedo, Fabio Fuentes-Gandara

was edited for proper English language, grammar, punctuation, spelling, and overall style by one or more of the highly qualified native English speaking editors at AJE.

This certificate was issued on **July 30, 2020** and may be verified on the AJE website using the verification code **8AFB-3C10-7F91-9BE3-56A0**.

Neither the research content nor the authors' intentions were altered in any way during the editing process. Documents receiving this certification should be English-ready for publication; however, the author has the ability to accept or reject our suggestions and changes. To verify the final AJE edited version, please visit our verification page at aje.com/certificate. If you have any questions or concerns about this edited document, please contact AJE at support@aje.com.

AJE provides a range of editing, translation, and manuscript services for researchers and publishers around the world. For more information about our company, services, and partner discounts, please visit aje.com.
