# Supplementary material for: Data on the diagnosis of the management of the primary waste from electrical and electronic equipment in health care institutions in Barranquilla, Colombia
Source: Data Brief. 2020 Aug 27;32:106236. doi: 10.1016/j.dib.2020.106236 (PMC7476238; doi:10.1016/j.dib.2020.106236)
Supplement: Supplementary file 4 [file mmc4.pdf]

EVALUATION OF THE INTEGRAL GESTION OF WASTE ELECTRICAL AND ELECTRONIC EQUIPMENT (WEEE) PRESENT IN HEALTH CARE INSTITUTIONS IN BARRANQUILLA-COLOMBIA.

Waste from electronic and electronic equipment is a complex mixture of many materials that are discarded and/or discarded, some of which as scarce and valuable raw materials can also be recovered for future use. However, these contain dangerous elements or compounds, which, while not causing problems during their use, become a considerable danger when released into the environment, it is therefore essential to make a proper disposal for such waste.

The aim of the survey presented below is to analyses the management of waste electrical and electronic equipment (WEEE) in the health care institutions of Barranquilla. The survey is focused on diagnostic and procedural teams considering their life cycle up to their final disposal.

It is clarified that the information submitted by each of the institutions will not be publicly disclosed and the analysis of the results will be worked as the overall averages obtained in each of the questions.

1. Name of the health care institution:

____

2. Indicate whether the HCI is public or private:

☐ Public ☐ Private

3. Main address:

____

4. Select whether the HCI has only one site or multiple sites

☐ Unique ☐ Multiple

4.1. If your answer was multiple sites, indicate whether the information you are providing is from your site or from all sites. Mention Them.

____

____

____

5. Charge of the personnel tested:

____

6. What type of electrical and electronic medical diagnostic equipment and/or procedures do you use?

____

____

____

The following list includes equipment that has direct contact with patients, in order to check if they can be treated for later use. Select the equipment you consider:

Hospital and Clinic Equipment:

☐ Defibrillator	☐ Wrist monitor	☐ Centrifuges
☐ Electrocardiograph	☐ Digital thermometer	☐ Freezers
☐ X-ray scanner	☐ Medical scales	☐ Doppler fetal monitor
☐ Medical lamps	☐ Anesthetic vaporizers	☐ Ultrasound machine
☐ Anesthesia machines	☐ Hematological equipment	☐ Incubators

☐ Vital signs monitor	☐ Autoclave	☐ Microscope
☐ Pulsioximeter (Pulse oximeter)	☐ Electrosurgery	☐ Photoceated lamp
☐ Infusion system	☐ Mechanical ventilator	☐ Photoceated lamp o curing light
☐ Suction unit	☐ Serological bath	☐ Radiology equipment

Other(s):

____

Dental equipment:

☐ Dental unit	☐ Curing lamp	☐ Dental compressor
☐ High-low speed handpiece	☐ Dental ultrasound machine	☐ Apical localizator
☐ Dental and panoramic radiology	☐ saliva and blood suction equipment	☐ Dental amalgamator
☐ Autoclave	☐ Curing light	☐ Dental anesthesia

Other(s):

____

Ophthalmological equipment

☐ Tonometer	☐ Lensometer	☐ Keratometry
☐ Indirect ophthalmoscope	☐ Slit Lamp	☐ Retinoscope

☐ Ophthalmoscope	☐ Ophthalmologic projector	☐ Pupilometer

Other(s):

____

7. What type of waste from Non-Biomedical electrical and electronic equipment are generated?

Select the equipment that you consider. Please keep in mind that in the option “Other” you can place the WEEE that you could not dispose of, since those are not listed in a post-consumer plan.

☐ Computer	☐ Devices with screens	☐ Roof lamps
☐ Table lamps and LEDs	☐ Medical lamps	☐ Diagnosis equipment
☐ Domestic appliances	☐ Electrical tools	☐ Other

Other:

____

8. Do you know the Colombian legal regulations for the WEEE management generated in this type of health care institutions?

☐ YES

☐ NO

☐ I DO NOT KNOW

8.1. Estimate the amount of WEEE-type waste produced in the institution, and in the box below, mention the names of the waste of this type that are most frequently generated.

☐ 0 – 20 kg/year	☐ 21 – 40 kg/year	☐ 41 – 60 kg/year

☐ 61 – 80 kg/year	☐ 81 – 100 kg/year	☐ 101 – 120 kg/year
☐ 121 – 150 kg/year	☐ Higher than 150 kg/year	

9. Do you have a collection or storage center for WEEE?

☐ YES

☐ NO

☐ I DO NOT KNOW

10. How do you store WEEE?

☐ Containers	☐ Open air	☐ Plastic bags
☐ Cardboard boxes	☐ Special plastic	☐ I do not know

Other:

____

11. What kind of difficulties have you had in storing this waste?

You can select various options according to your case.

☐ Little space to store	☐ Carrying equipment	☐ Size of the equipment
☐ Brittleness of equipment	☐ None	

Other:

____

12. What is the final disposition of the stored equipment?

You can select various options according to your case.

☐ Return to suppliers	☐ Part reuse	☐ Donation
☐ post-consumption plan	☐ Equipment sales	☐ Reciclying
☐ Cleanliness operator	☐ Scrapping	☐ No final disposition

Other:

____

13. What is the amount of equipment is currently stored and is destined for final disposal?

☐ From 1 to 10	☐ From 11 to 20	☐ From 21 to 30
☐ From 31 to 40	☐ From 41 to 50	☐ Not sure
☐ Currently empty		

Other:

____

14. Apart from WEEE, what other types of elements that are solid waste, do you consider have difficulties for their final disposal or could be used?

15. What other types of equipment do you consider could be managed as WEEE within the Post-consumption plans?
